# Sphingolipid de novo biosynthesis is essential for intestine cell survival and barrier function

**DOI:** 10.1038/s41419-017-0214-1

**Published:** 2018-02-07

**Authors:** Zhiqiang Li, Inamul Kabir, Gladys Tietelman, Chongmin Huan, Jianglin Fan, Tilla Worgall, Xian-Cheng Jiang

**Affiliations:** 1 Department of Cell Biology, State University of New York, Downstate Medical Center, Brooklyn, NY 11203 USA; 2 Molecular and Cellular Cardiology Program, VA New York Harbor Healthcare System, Brooklyn, NY 11209 USA; 3 Department of Molecular Pathology, University of Yamanashi, Yamanashi, Japan; 4 Department of Medicine, Columbia University, New York, NY 10032 USA

## Abstract

Serine palmitoyltransferase (SPT) is the rate-limiting enzyme for sphingolipid biosynthesis. SPT has two major subunits, SPTLC1 and SPTLC2. We previously found that liver *Sptlc2* deficiency in early life impairs the development of adherens junctions. Here, we investigated the role of *Sptlc2* deficiency in intestine. We treated *Sptlc2*-Flox/villin-Cre-ER^T2^ mice with tamoxifen (days 1, 2, and 3) to ablate *Sptlc2* specifically in the intestine. At day 6 after tamoxifen treatment, *Sptlc2*-deficient mice had significantly decreased body weight with concurrent diarrhea and rectal bleeding. The number of goblet cells was reduced in both large and small intestine of *Sptlc2*-deficient mice compared with controls. *Sptlc2* deficiency suppressed the level of mucin2 in the colon and increased circulating lipopolysaccharides, suggesting that SPT activity has a housekeeping function in the intestine. All *Sptlc2*-deficient mice died 7–10 days after tamoxifen treatment. Notably, supplementation with antibiotics and dexamethasone reduced lethality by 70%. We also found that colon specimens from patients with inflammatory bowel diseases had significantly reduced *Sptlc2* expression, SPTLC2 staining, and goblet cell numbers. SPT activity is crucial for intestinal cell survival and barrier function.

## Introduction

SPT is the first and rate-limiting enzyme involved in sphingolipid biosynthesis^1^. The mammalian SPT holoenzyme is primarily a heterodimer composed of two protein subunits, SPTLC1 (53 kDa) and SPTLC2 (63 kDa), which share 20% amino acid sequence identity^2,3^. However, studies indicate the existence of a third subunit, SPTLC3, which has 68% identity to SPTLC2^4^. Additionally, two low-molecular-weight proteins, ssSPTa and ssSPTb, enhance the activity and confer distinct acyl-CoA substrate specificities to mammalian SPT, similar to the yeast Tsc3p subunit^5^. A relatively recent discovery indicated that yeast ORM (orosomucoid) 1/ORM2 proteins also associate with and negatively regulate SPT activity^6^, thus adding another layer of complexity. Based on this new observation, a new term “SPOTS complex” (SPTLC1/2, ORM1/2, Tsc3, Sac1) was proposed^6^. These studies provide a starting point for investigating how protein and lipid synthesis is coordinated during cell membrane biogenesis.

Perturbations in SPT activity have been linked to diseases. Specific mutations identified in *Sptlc1* or *Sptlc2* cause a rare genetic disorder called hereditary sensory and autonomic neuropathy type 1^7–9^. The lack of *Sptlc1* or *Sptlc2* in mice causes embryonic lethality^10^. SPTLC1/SPTLC2 binds the cell polarity factor Par3 and modulates monocyte chemotaxis^11^. Park et al.^12^ and we^13^ reported that treatment of *Apoe* knockout (KO) mice with myriocin, a highly selective inhibitor of SPT activity, decreases plasma sphingomyelin levels (via oral administration) and atherosclerosis (via intraperitoneal injection). However, myriocin often causes severe gastrointestinal side-effects^14^, but the basis is unknown. We recently reported that liver-specific *Sptlc2* deficiency in mice during early life impairs hepatocyte polarity through decreasing the levels of membrane factors that are involved in the formation of adherens junctions, thus promoting liver tumorigenesis^15^. We proposed an important role for SPT activity in establishing cell polarity and tissue integrity. As is the case for hepatocytes, enterocyte polarity is essential for intestinal functions. Among these functions, intestinal barrier function is the most important one.

Recent studies have clearly demonstrated the role of gut microbiota in health and chronic gastrointestinal disease^16^, but our knowledge of gut sphingolipid biosynthesis and barrier function remains incomplete. Emerging evidence suggests that sphingolipid metabolism contributes to the development of inflammatory bowel disease (IBD). Sakata et al.^17^ demonstrated that blocking the generation of ceramides with the Sphingomyelinase inhibitor hinders mouse colitis. Fischbeck et al.^18^ showed that increasing ceramides in the gut by supplying mice with dietary sphingomyelins, a precursor of ceramides, and aggravates mouse colitis. Wang et al.^19^ found that alkaline ceramidase 3 deficiency aggravates colitis and colitis-associated tumorigenesis. Notably, intestinal permeability is influenced by membrane sphingolipids^20^.

To further address the relationship between sphingolipid biosynthesis and gastrointestinal diseases, we created a mouse line in which *Sptlc2* could be inducibly knocked out in the intestine to evaluate the impact of SPT activity on intestinal barrier function. We hypothesized that *Sptlc2* deficiency in the intestine impairs cell polarity through reducing sphingolipid levels in the plasma membrane; the consequent change in gut permeability then allows previously immune-transparent microbes to become targeted by the host immune system. However, what we found was that the blockage of sphingolipid de novo synthesis has a dramatic impact on intestinal cell survival and barrier function.

## Results

### Preparation of inducible intestine-specific *Sptlc2* KO mice

We prepared intestine-specific non-inducible *Sptlc2* KO mice by crossing Villin-Cre transgenic mice with *Sptlc2*-Flox mice. No homozygous *Sptlc2* KO mice could be obtained after screening more than 100 offspring, so we chose an inducible approach (Supplementary Figure S1A).

**Fig. 1:**
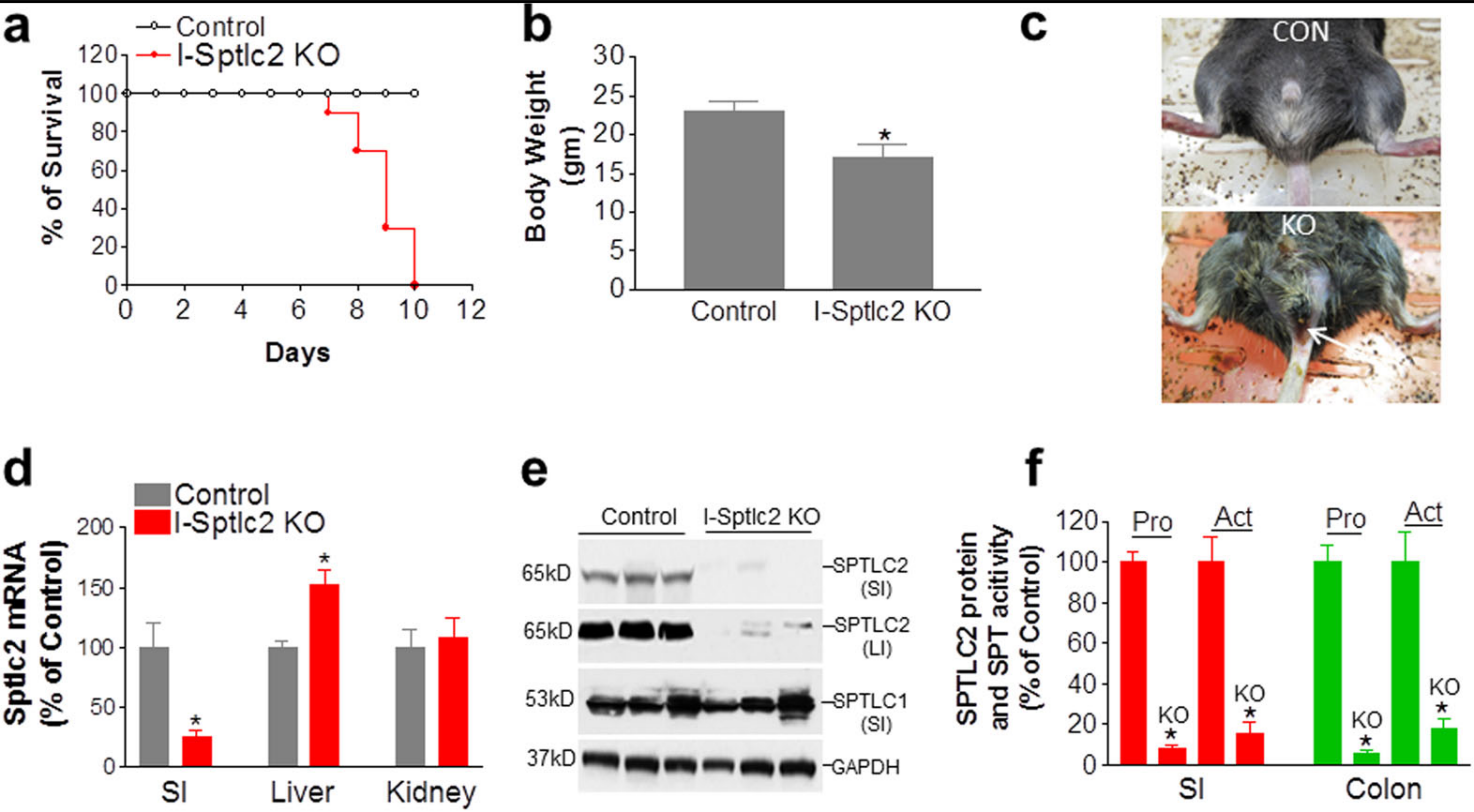
Characterization of intestine-specific inducible *Sptlc2* KO mice. *Sptlc2*-flox/Villin-Cre-ER^T2^ mice and controls were injected three times with tamoxifen (0.5 mg per mouse, intraperitoneally). **a** Kaplan–Meier survival curve for intestine-specific Sptlc2-deficient mice, *n* = 20. **b** Mouse body weight data on day 6 after tamoxifen treatment. **c** Image of a Sptlc2 mutant mouse with diarrhea and rectal bleeding. **d** SPTLC2 mRNA in different tissues was measured by real-time PCR on day 6 after tamoxifen treatment. **e** Western blotting for SPTLC2 and SPTLC1. **f** Quantification of SPTLC2 protein and activity in small intestine (SI) and large intestine (LI). I-Sptlc2 KO, intestine specific *Sptlc2* KO mice. SI, small intestine. Values represent the mean ± SD, *n* = 5, *P < 0.01

We first prepared *Sptlc2*-Flox mice. We then crossed these mice with heterozygous Villin-Cre-ER^T2^ mice to obtain two lines of mice: *Sptlc2*-Flox (heterozygous)/Villin-Cre-ER^T2^ (heterozygous) and *Sptlc2*-Flox (heterozygous) mice. We then crossed these two lines of mice to obtain experimental and control mice: *Sptlc2*-Flox (homozygous)/Villin-Cre-ER^T2^(heterozygous) and WT mice (Supplementary Figure S1B). The genetic background of all these mice was C57BL/6. To prepare intestine-specific *Sptlc2*-deficient mice, tamoxifen (0.5 mg per mouse), dissolved in corn oil, was injected intraperitoneally into the male mice on days 1, 2, and 3. Tamoxifen-treated *Sptlc2*-Flox mice were used as controls. The tamoxifen-treated *Sptlc2*-Flox/Villin-CreER^T2^ mice died within 7–10 days after treatment, but this was not the case for the control mice (*Sptlc2*-Flox) (Fig. 1a). We thus characterized the mice on day 6 after tamoxifen treatment. The *Sptlc2*-deficient mice had reduced body weight (Fig. 1b, Supplementary Figure S2A) and food consumption (Supplementary Figure S2B), as well as diarrhea and rectal bleeding (Fig. 1c). The dying mice had significantly lower body temperature (Supplementary Figure S2C). SPTLC2 mRNA level was decreased by 80% in the intestine but not in other tissues (Fig. 1d). We then measured SPTLC2 protein level in homogenates of both small intestine and colon from *Sptlc2*-deficient mice and found that it was decreased by 95 and 92%, respectively, compared with controls (Fig. 1e, f); SPT activity was also dramatically reduced in the small intestine (by 90%) and colon (by 85%) (Fig. 1f). Similar results were obtained with female mice (data not shown). Notably, there was no change in the overall level of SPTLC1 protein (Fig. 1e). We also measured mRNA levels of ORMDL1–3, ssSPTa, and ssSPTb in the colon. We found that all of them were significantly reduced (Supplementary Figure S3A and B).

### Intestine-specific *Sptlc2* deficiency disrupts intestinal barrier function

**Fig. 2:**
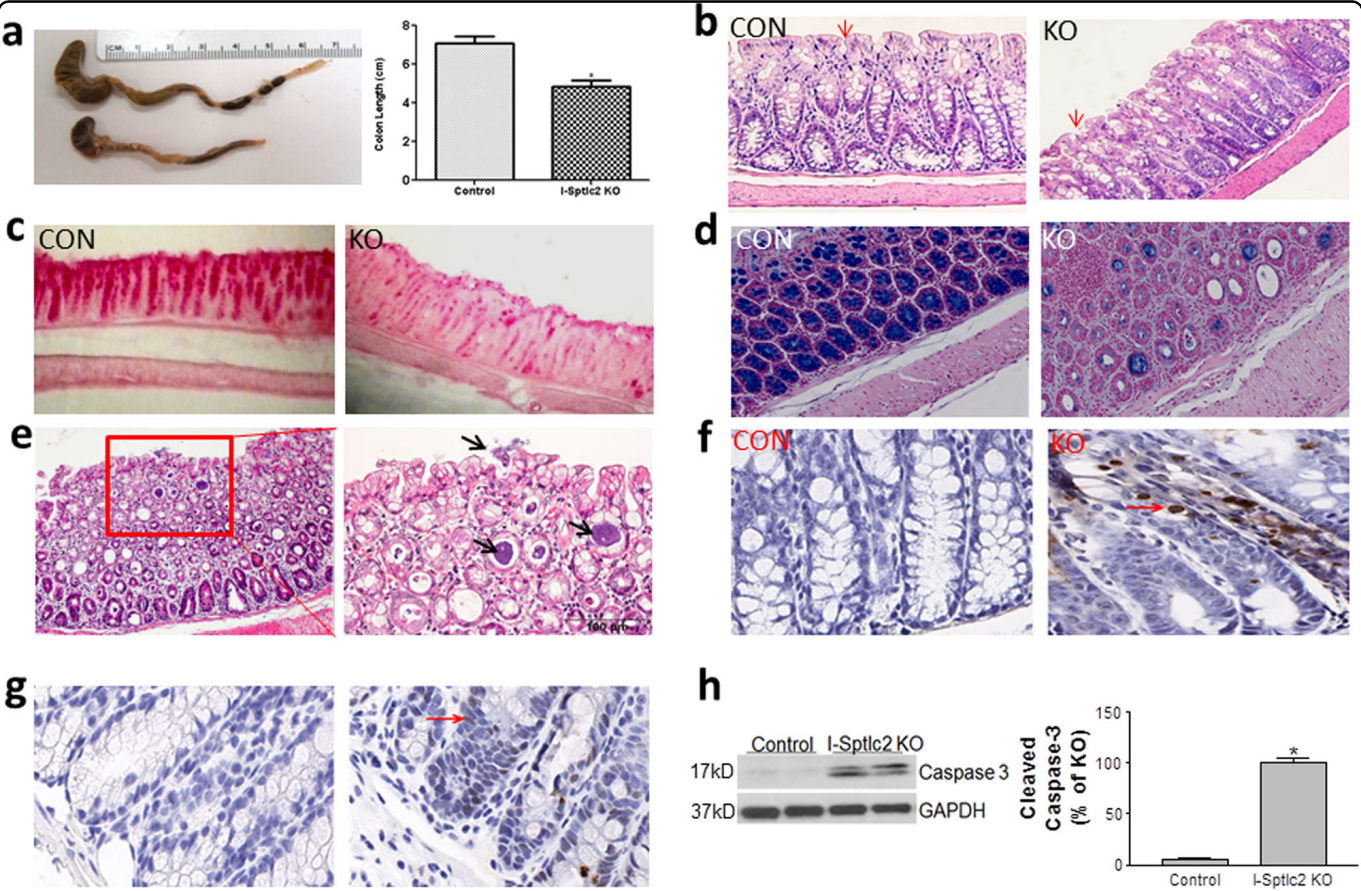
Effect of *Sptlc2* deficiency on the colon. **a** Images depicting *Sptlc2* KO mouse colon length and quantification at day 6 after tamoxifen treatment. **b** H&E staining of the colon. Red arrows indicate the top part of crypts. **c** Goblet cells were stained with periodic acid-Schiff. **d** Goblet cells were stained with Alcian Blue. **e** Image of H&E-stained Sptlc2-deficient colon at higher magnification. Black arrows indicate bacterial clusters at the top and in the middle of the mucosa). **f** TUNEL staining of mouse colon. **g** Immunostaining for cleaved caspase-3 in the colon. **h** Western-blot fluorogram and quantification of cleaved caspase-3 in the colon. I-Sptlc2 KO, intestine specific Sptlc2 KO mice. Values represent the mean ± SD, *n* = 5, *P < 0.01

We measured sphingolipid levels in the plasma membrane of colon cells and found that *Sptlc2*-Flox/Villin-CreER^T2^ mice had significantly smaller amounts of all tested ceramides and sphingomyelins (Table 1) after tamoxifen treatment, but no changes were observed for sphingosine-1-phosphate (1.48 ± 0.32 vs. 1.17 ± 0.10 ng/mg protein). Necropsy revealed that the *Sptlc2*-deficient mice had a shorter colon compared with controls (Fig. 2a), a similar phenotype observed in inducible intestine-specific Kindlin 1 and 2 KO mice^21^.

**Table 1:** Sphingolipid levels on the plasma membrane of intestine cells

Mice	C16:0	C18:0	C18:1	C24:1	C24:0	C20:0	C22:0
(ng/mg protein)							
**Colon**							
Sphingomyelin							
WT	3908 ± 205	1092 ± 90	71 ± 5	893 ± 64	–	–	–
*Sptlc2* KO	1872 ± 1353*	361 ± 226*	22 ± 15*	314 ± 128*	–	–	–
Ceramide							
WT	546 ± 42	67 ± 10	–	132 ± 27	38 ± 2	30 ± 10	73 ± 7
*Sptlc2* KO	240 ± 143*	27 ± 19*	–	56 ± 21*	21 ± 11*	14 ± 9*	34 ± 19*
**Small intestine**							
Sphingomyelin							
WT	6157 ± 510	1701 ± 230	109 ± 16	1398 ± 160	–	–	–
*Sptlc2* KO	1919 ± 254**	203 ± 37**	16 ± 6**	221 ± 23**	–	–	–
Ceramide							
WT	903 ± 126	106 ± 16	–	217 ± 35	62 ± 8	48 ± 6	115 ± 14
*Sptlc2* KO	188 ± 46**	16 ± 3**	–	43 ± 3**	23 ± 3**	11 ± 2**	27 ± 4**

Values: mean ± SD; *n* = 5

*WT* wild type

*P  < 0.05; **P  < 0.01

We next assessed colon morphology via staining with hematoxylin and eosin (H&E; Fig. 2b), periodic acid-Schiff (Fig. 2c), and Alcian Blue (Fig. 2d), which revealed that the ablation of *Sptlc2* in intestine dramatically reduced the number of goblet cells in the colon compared with controls. We noticed that bacteria had infiltrated the mucosa of the *Sptlc2*-deficient mouse colon (Fig. 2e) and that the colon had disorganized crypts of Lieberkühn (Fig. 2b). Importantly, both TUNEL (terminal deoxynucleotidyl transferase-mediated dUTP nick end labeling) and immunostaining for cleaved caspase-3 clearly indicated that *Sptlc2* deficiency aggravated apoptosis in the colon compared with the control group (Fig. 2f, g), and this was confirmed by western blotting for cleaved caspase-3 (Fig. 2h). We immunostained T and B lymphocytes with CD4 and B330 antibodies, respectively. We found both lymphocytes were significantly accumulated in *Sptlc2* deficient colon (Supplementary Figure S4A and B).

**Fig. 3:**
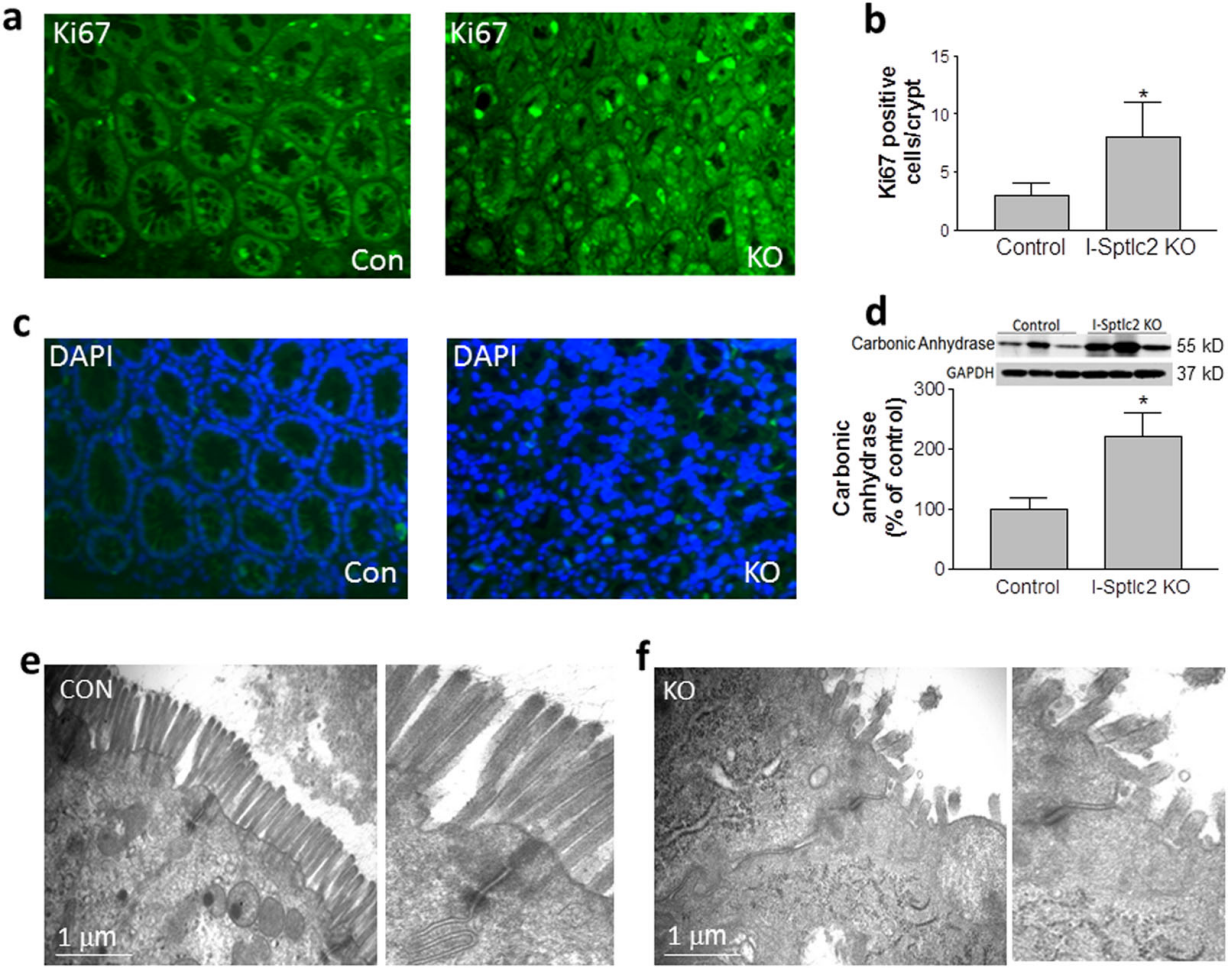
Effect of *Sptlc2* deficiency on colon cell differentiation and proliferation. **a** Immunostaining for Ki67. **b** Quantification of Ki67 in crypts. **c** DAPI staining to indicate nuclei. **d** Western-blot fluorogram and quantification of carbonic anhydrase in the colon. Values represent the mean ± SD, *n* = 5, *P < 0.01. **e**–**f** Electron microscopic image of mouse colon. All images are representative of five fields each. CON control, KO intestine-specific Sptlc2 knockout

We also found that significantly more *Sptlc2* deficient colon cells underwent proliferation (Fig. 3a, b) and differentiation, as suggested by western blotting for carbonic anhydrase^22^ (Fig. 3d). The disorganization of the crypts of Lieberkühn was also confirmed by staining with DAPI (4′,6-diamidine-2′-phenylindole dihydrochloride) (Fig. 3c).

We next visualized mouse colon with electron microscopy and found far fewer microvilli in the *Sptlc2*-deficient colon and that the microvilli were shorter and disorganized compared with controls (Fig. 3e, f), suggesting that inhibition of SPT activity in the intestine impaired cell integrity and structure.

**Fig. 4:**
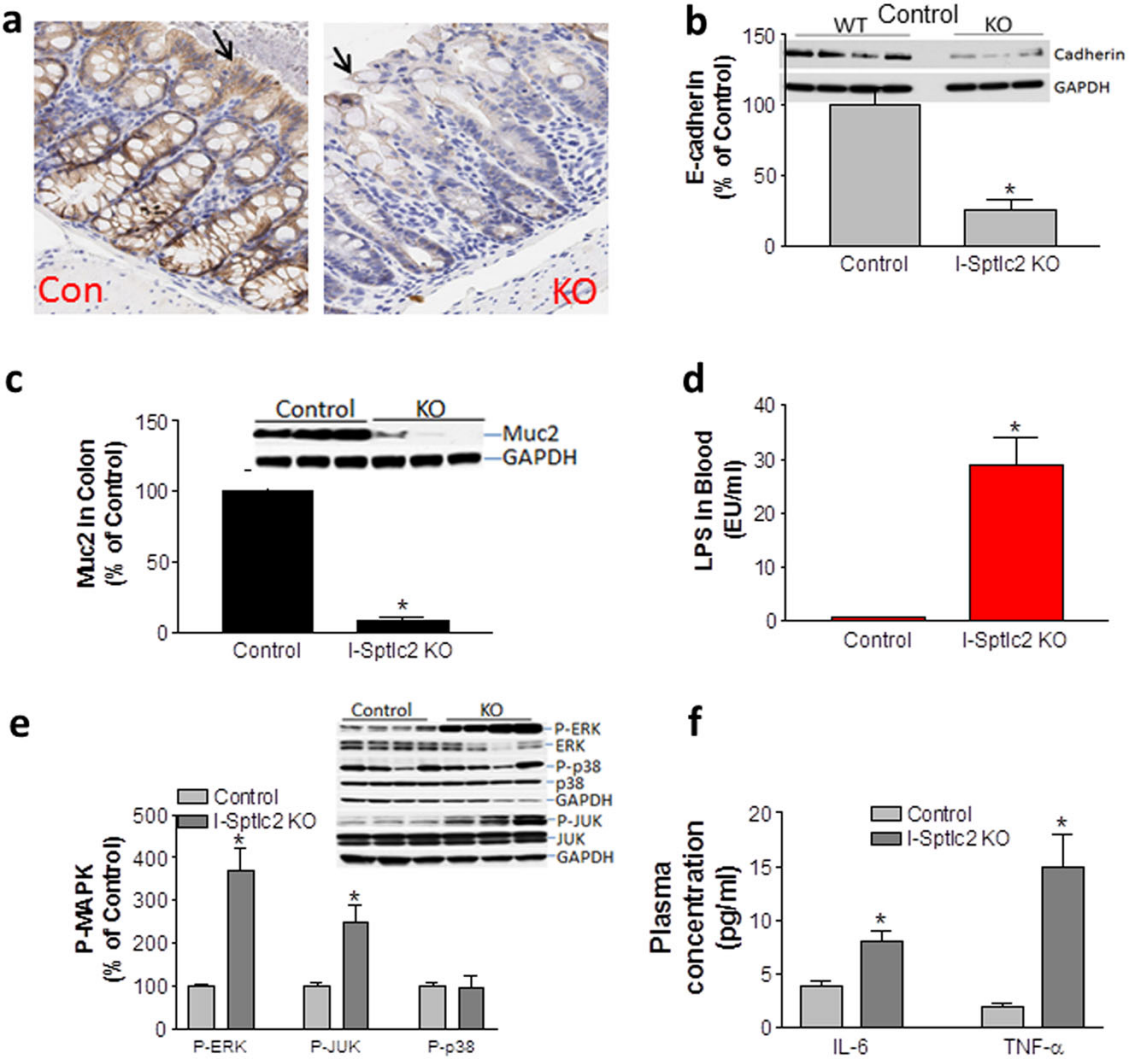
Effect of *Sptlc2* deficiency on cadherin, mucin2, and MAP kinases in the colon. **a** Immunostaining for E-cadherin in the colon. Black arrows indicate the top part of crypts. **b** Western-blot fluorogram and quantification of E-cadherin in the colon. **c** Western-blot fluorogram and quantification of mucin2. **d** LPS level in mouse plasma. **e** Western-blot fluorogram and quantification of phospho-ERK and total ERK, phospho-JNK and total JNK, and phospho-p38 and total p38 in the colon of Sptlc2-deficient and control mice. **f** Plasma IL-6 and TNF-α measurement. Values represent the mean ± SD, *n* = 5, *P < 0.01. CON control, KO intestine-specific Sptlc2 knockout

Immunohistochemical analysis revealed that *Sptlc2* ablation caused a dramatic reduction of E-cadherin, an important plasma-membrane protein for adherens junctions^23^ (Fig. 4a). This was confirmed by western blotting for cadherin (Fig. 4b).

We next sought to measure mucin2 level in the *Sptlc2*-deficient colon. Mucin2 is the major protein secreted by goblet cells, which provide a barrier function for the gut^24^. We found that mucin2 was reduced by 90% compared with control mice (Fig. 4c), suggesting a disruption of intestinal barrier function. We also found that the KO mice had a very high level of plasma lipopolysaccharides (LPS) compared with control mice (Fig. 4d). This high level of plasma LPS may have caused acute endotoxinemia, which would explain the lethal phenotype. We then measured the colon MAPK levels, which mediates inflammation. We found that *Sptlc2* deficiency significantly increased the phosphorylation/activation of ERK and JNK but not p38 (Fig. 4e) in the colon compared with control mice. Moreover, we found that the KO mice have significantly higher IL-6 and TNFα in the circulation (Fig. 4f).

**Fig. 5:**
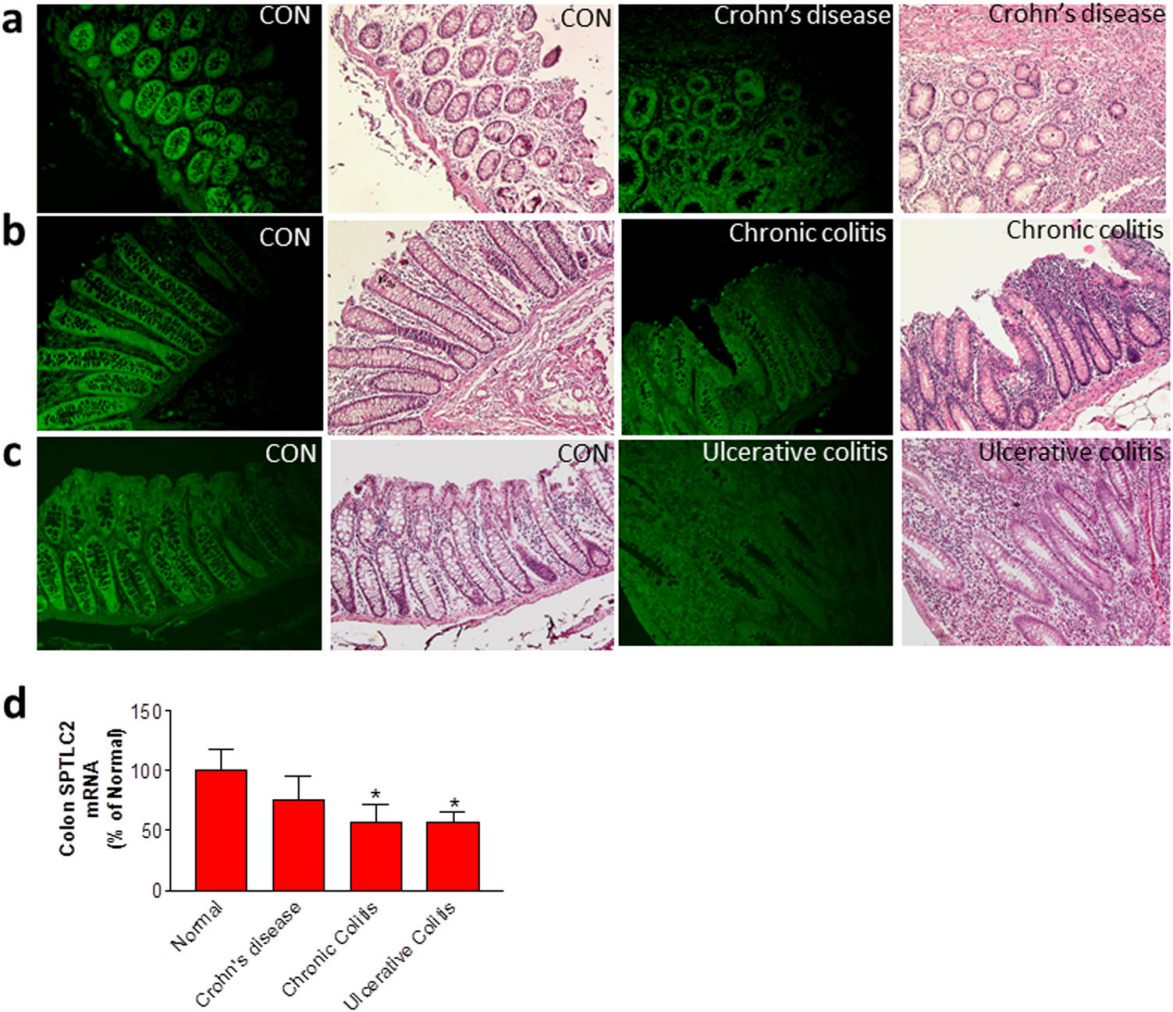
Relationship between human inflammatory bowel diseases and SPTLC2. We utilized a SPTLC2-specfic antibody and H&E stained colon samples from patients with Crohn’s disease (**a**), chronic colitis (**b**), and ulcerative colitis (**c**), and compared the images with that of the control (CON). (**d**) We obtained human Crohn’s and colitis tissue qPCR array from QriGene Technologies, Inc. (Cat# CCRT101), and then we did real-time PCR for SPTLC2 mRNA. Values represent the mean ± SD, control group *n* = 4, Crohn’s disease group *n* = 6, chronic colitis group *n* = 4, ulcerative colitis group *n* = 14, *P < 0.05

We next evaluated the relationship between SPT activity and human inflammatory bowel diseases. We utilized a SPTLC2-specific antibody to immunostain colon samples that were obtained from human patients with Crohn’s disease, chronic colitis, and ulcerative colitis, as well as control. Colons afflicted with the three disease types had much less intense SPTLC2 staining and fewer goblet cells and had greater lymphocyte infiltration compared with controls (Fig. 5a–c). Moreover, all diseased colons expressed less SPTLC2 mRNA, although this difference was not statistically significant for Crohn’s disease (Fig. 5d). Thus, sphingolipid biosynthesis has a direct impact on human inflammatory bowel diseases.

**Fig. 6:**
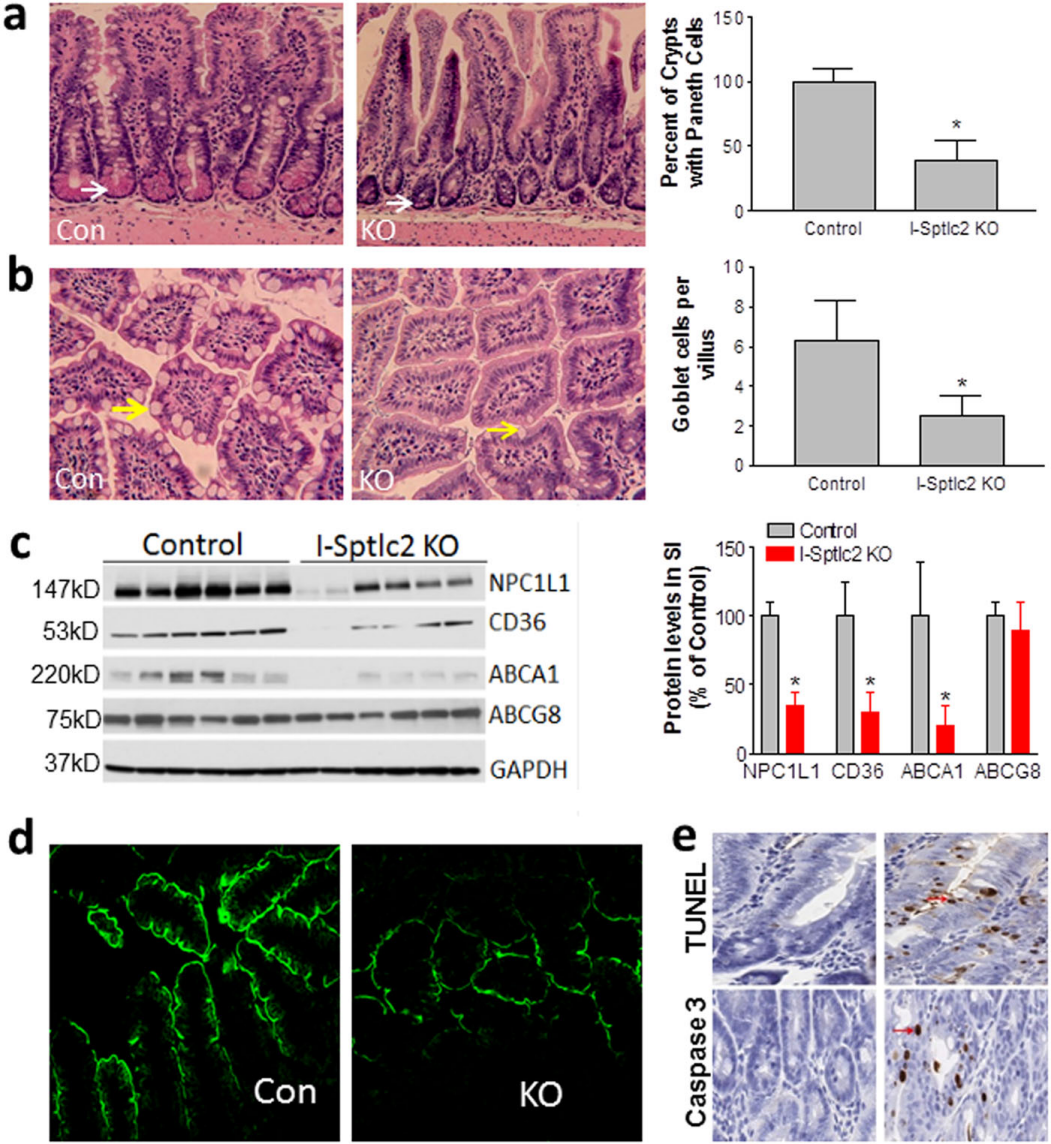
Effect of *Sptlc2* deficiency on the small intestine. **a** H&E staining of Paneth cells and quantification in jejunum. White arrow indicates Paneth cells. **b** H&E staining of and goblet cells and quantification in jejunum. Yellow arrow indicates goblet cells. Values represent the mean ± SD, *n* = 30, *P < 0.001. **c** Western-blot fluorogram and quantification of NPC1L1, CD36, ABCA1, and ABCG8 in the plasma membrane of primary enterocytes. Values represent the mean ± SD, *n* = 5, *P < 0.01. **d** Immunostaining for NPC1L1 in the jejunum. **e** TUNEL and immunostaining for cleaved caspase-3 in the colon. Red arrows indicate apoptotic cells. All images are representative of five fields each

We next sought to assess sphingolipid subspecies on the plasma membrane of enterocytes from the small intestine. After tamoxifen treatment, the *Sptlc2*-deficient mice had significantly decreased amounts of all tested ceramides and sphingomyelins (Table 1) but not sphingosine-1-phosphate (2.09 ± 0.68 vs. 1.69 ± 0.21 ng/mg protein). These changes could also have an impact small intestine morphology and pathology. Indeed, we found a significant reduction of both Paneth cells (Fig. 6a) and goblet cells (Fig. 6b) in the *Sptlc2*-deficient small intestine compared with the control. Next, we used western blotting to measure the levels of Niemann-Pick C1-like 1 (NPC1L1), CD36, ABCG8, and ABCA1, revealing a significant decrease in NPC1L1, CD36, and ABCA1 (62, 65, and 70, respectively; *P* < 0.01); the level of ABCG8 was not significantly affected (Fig. 6c). We also used immunofluorescence staining to assess the level of apical-membrane NPC1L1, which is involved in cholesterol uptake, and found that it was dramatically decreased in the small intestine of the *Sptlc2* KO mice compared with the control (Fig. 6d). These results suggested that changes in the amounts of plasma-membrane sphingolipids can affect their localization on the apical membrane of enterocytes. Further, we found that the KO mice significantly reduced cholesterol absorption compared with controls (Supplementary Figure S5). Moreover, both TUNEL and immunohistochemical analysis for cleaved caspase-3 clearly indicated that *Sptlc2* deficiency also promoted apoptosis in the small intestine compared with the control group (Fig. 6e).

We also noticed that the *Sptlc2*-deficient mice had a significantly smaller spleen, fewer splenocytes (Supplementary Figure S6A), and lower levels of circulating white blood cells and lymphocytes (Supplementary Figure S6B). Moreover, compared with control mice, the *Sptlc2*-deficient mice exhibited substantially more inflammation in the lung, with more numerous infiltrated lymphocytes, and thicker septa (Supplementary Figure S6C), a typical phenotype of severe lung inflammation.

### Intestinal *Sptlc2* deficiency-mediated lethality can be rescued

**Fig. 7:**
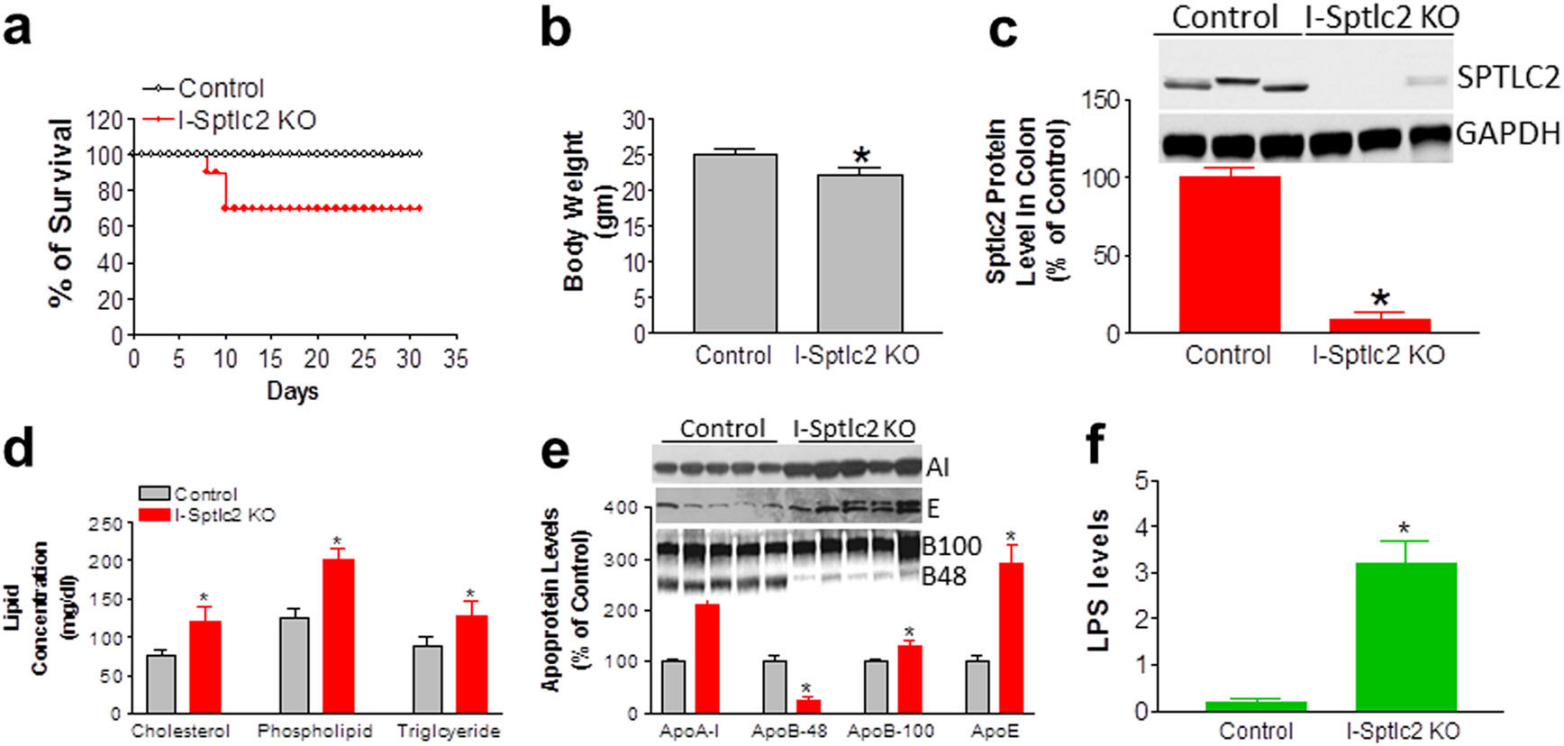
Intestinal *Sptlc2* deficiency–mediated lethality can be rescued. **a** Kaplan–Meier survival curve for intestine-specific Sptlc2-deficient mice after rescue with antibiotics and dexamethasone, *n* = 20. **b** Body weight measurement at day 16. **c** Western-blot fluorogram and quantification of SPTLC2 in the colon at day 16. **d** Measurement of total plasma cholesterol, phospholipids, and triglycerides at day 16. **e** Western-blot fluorogram and quantification of plasma apolipoprotein levels at day 16. Plasma (0.2 µl) was separated by gradient (4–15% acrylamide) SDS-PAGE and subjected to western blotting with polyclonal antibodies against apoA-I, apoB, and apoE. **f** Plasma LPS levels at day 16. Values represent the mean ± SD, *n* = 5, *P < 0.05

The observed phenotypes suggested that the *Sptlc2* deficiency-related lethality is related to the impairment of intestinal barrier function. Hence, we conjectured that, prior to tamoxifen administration, pre-treating the mice with low absorbable antibiotics to cleanse the existing bacterial flora in the gut and with dexamethasone to induce an anti-inflammatory response could rescue the mice. Indeed, treatment of mice with antibiotics and dexamethasone rescued 70% of the mice (Fig. 7a) and no death was observed after day 10, although the average body weight of the *Sptlc2* KO mice was still less than that of the control at day 16 (Fig. 7b). We then measured SPTLC2 protein level after rescue (at day 16) and again found that the KO mice had dramatically decreased SPTLC2 in the colon (90%; Fig. 7c).

We also measured plasma lipid levels in these rescued mice and found that these mice had significantly increased levels of plasma cholesterol (55%, *P* < 0.01), total phospholipids (60%, *P* < 0.01), and triglycerides (40%, *P* < 0.01) compared with controls (Fig. 7d). The female KO mice had a very similar phenotype (data not shown). Plasma apolipoprotein levels in *Sptlc2* KO and control mice were assessed with reducing SDS-PAGE, which revealed a significant increase in plasma apoA-I (*P* < 0.001) and apoE (*P* < 0.001) (Fig. 7e). We also found a 70% reduction in apoB48 (*P* < 0.001), suggesting a defect in chylomicron production, and a small but significant 22% induction in apoB100 (*P* < 0.05) (Fig. 7e), suggesting enhanced production of very low–density lipoproteins in the liver.

We hypothesized that the observed lipid profile of *Sptlc2* KO mice could be due to the effect of residual plasma LPS on the liver, because high plasma LPS correlates with hyperlipidemia^25^. Indeed, although treatment with antibiotics and dexamethasone rescued 70% of the *Sptlc2* KO mice, the residual LPS in the circulation was still significantly higher than in controls (3.2 ± 0.8 vs. 0.6 ± 0.1 EU/ml; Fig. 7f).

## Discussion

In this study, we prepared intestine-specific inducible *Sptlc2*-deficient mice by crossing *Sptlc2*-Flox mice with Villin-Cre-ER^T2^ transgenic mice. We demonstrated that disruption of *Sptlc2* in the intestine resulted in: (1) a significant decrease of ceramide and sphingomyelin in the plasma membrane of colon and small-intestine cells; (2) a promotion of intestinal cell apoptosis and death, and impairment of intestinal barrier function; and (3) accumulation of LPS in the circulation, presumably causing lethality. Moreover, pretreatment with antibiotics and dexamethasone could rescue intestine-specific *Sptlc2* deficiency–mediated lethality. Importantly, intestine-specific *Sptlc2*-deficient mice can mimic certain phenotypes observed in human inflammatory bowel diseases, such as Crohn’s disease, chronic colitis, and ulcerative colitis. To our knowledge, this is the first study linking sphingolipid de novo biosynthesis with intestine cell survival and barrier function.

Our key finding is that SPT activity is a critical factor for maintaining intestine integrity. It is most likely that the barrier defects observed in *Sptlc2* KO mice are due to epithelial damage. *Sptlc2* depletion in the intestine led to the death of all mice within 7–10 days after the administration of tamoxifen. Before death, all *Sptlc2*-deficient mice exhibited shivering, thus mimicking the phenotype of LPS-induced acute toxicity. These results suggest that *Sptlc2* deficiency-mediated epithelium cell death impairs intestinal barrier integrity that is maintained by the mucus layer (secreted by goblet cells) and by antimicrobial agents (secreted by Paneth cells)^26,27^, thus promoting the infiltration of gut bacteria into the circulation.

The essential requirement for sphingolipid de novo biosynthesis in intestine cells consists with some other cell types. We found that liver *Sptlc2* deficiency in early life impairs the development of hepatocyte polarity, resulting in severe jaundice and tumorigenesis^15^. Sphingolipid biosynthesis is also required for adipocyte survival, because adipocyte-specific *Sptlc1* (another major subunit of SPT) KO mice exhibited a striking age-dependent loss of adipose tissues^28^ and adipocyte-specific *Sptlc2* KO mice exhibited similar lipodystrophy phenotype^29^. The essential requirement for sphingolipid biosynthesis indicates that these cells are unable to acquire sufficient sphingolipids through alternative uptake and recycling pathways for cell survival.

The requirement for sphingolipid biosynthesis in intestine cells contrasts with some other cell types. We also found that an adult liver-specific *Sptlc2* KO mouse exhibited reduced ceramide and sphingomyelin in both liver and plasma, but it displayed no apparent adverse effect on the hepatocytes^30^. In line with this, macrophage-specific *Sptlc2* deficiency did not alter macrophage polarization and inflammatory capability^31^.

Which sphingolipid lacking, meditated by SPT deficiency, is responsible for the cell death? Sphingomyelin synthase 1 (*Sms1*) KO mice have been shown to have a severe lipodystrophy, with evidence of adipocyte cell death^32^. It was inferred that ceramide accumulation, due to the block in conversion to sphingomyelin, was responsible for the phenotype. However, adipocyte-specific *Sptlc2* deficiency results in the reduction of sphingolipids, including both sphingomyelin and ceramide, yields a similar lipodystrophy phenotype as that of *Sms1* deficiency^28^. We found in this study that both sphingomyelin and ceramide were decreased in intestine cells of our KO mice (Table 1). Thus, ceramide is not likely to play a role in cell death. The contribution of other sphingolipids to the phenotype was also considered. Sphingosine-1-phosphate, a sphingolipid that regulates processes such as inflammation^33^, could be another mediator. However, we did not observe a significant change of sphingosine-1-phosphate in the intestine. Collectively, our results together with others suggest that the reduction of sphingomyelin may be a potential cause of this shared phenotype. We found that *Sptlc2* deficiency reduced the levels of almost all sphingomyelins (Table 1), which are the major components of lipid rafts in the plasma membrane^34^. It is known that E-cadherin stabilization at cell–cell junctions requires raft microdomains^35,36^. Thus, *Sptlc2* deficiency-mediated reduction in the amounts of sphingomyelins could decrease E-cadherin in lipid rafts and could result in losing cell polarity^15^. Further studies are need for evaluating the relevance.

Inflammatory bowel diseases often involve environmental factors, infectious microbes, ethnicity, and a dysregulated immune system^37^. The genetic contribution is poorly understood^37,38^. Patients with an inflammatory bowel disease exhibit increased intestinal permeability, which favors bacterial/viral infections^39^. Consistent with this information, in most mouse models of colitis, recapitulation of the disease requires bacteria^40^. It is known that intestinal permeability is influenced by membrane sphingolipids^20^. However, the mechanism by which permeability becomes compromised is still controversial. On the one hand, treatment fumonisin B1 (a fungal inhibitor of ceramide synthase that leads to depletion of ceramide as well as complex glycosphingolipids) treatment induces a primary defect in barrier function, thereby increasing intestinal epithelial permeability^41^. In line with this observation, treatment of pigs with fumonisin B1 was found to significantly increase intestinal colonization by pathogenic *Escherichia coli*^42^. Moreover, dietary glycosphingolipids can enhance intestinal permeability by enhancing cell junctions^43^. On the other hand, sphingomyelinase-mediated generation of ceramide might perturb barrier function as seen in inflammatory bowel diseases^44^. The underlying cause(s) of this discrepancy between studies is unknown. It is possible that each of these studies was able to delineate only part of the overall mechanism. It is necessary to use an animal model to genetically deplete the pathway for sphingolipid de novo biosynthesis and then study the consequences. Therefore, in our present study, we created intestine-specific inducible *Sptlc2* KO mice and found that a deficiency of sphingolipid *de novo* biosynthesis in the intestine promoted cell apoptosis, damaged intestine integrity, and increased intestinal permeability (Fig. 5f); moreover, patients with an inflammatory bowel disease were found to have a significantly lower level of colon SPTLC2 mRNA and of SPTLC2 staining intensity (Fig. 6a–d).

Necropsy revealed that the *Sptlc2*-deficient mice had a shorter colon compared with controls (Fig. 2a). How is this remodeling possible when the mice only have a lifespan of 6–10 days after tamoxifen induction? We also puzzled by this observation. However, a most recent report also observed similar phenotype in three days after tamoxifen-induced villin-Cre-dependent intestinal deletion of Kindlin 1 and 2^21^. The authors observed that intestine specific deletion of kindlin 1 and 2 resulted in tight junction deletion and mucosal inflammation which was compatible with human ulcerative colitis^21^. We believe that shortened colon observed in our KO mice is related with intestinal cell death within 6–10 days after tamoxifen treatment.

It is well known that growth in epithelial cell proliferation and apoptosis correlates specifically to the inflammation activity of inflammatory bowel diseases^45–47^. In this study, we added one more evidence for this phenomenon. We found intestine *Sptlc2* deficiency causes more intestine cell apoptosis (Fig. 2f–h) and more proliferations (Fig. 3a, b). We believe that *Sptlc2* deficiency induces cell apoptosis which promotes the loss of gut epithelium. It might signal the stem cell proliferation and differentiation (Fig. 3d) in the crypt to replenish the dying enterocytes.

The mammalian SPT holoenzyme is primarily a heterodimer composed of two protein subunits, SPTLC1 and SPTLC2^2,3^ or SPTLC1 and SPTLC3^4^. SPTLC1 is not directly involved in the catalytic reaction; rather, it acts as an anchor that tethers SPTLC2 or SPTLC3 to the endoplasmic reticulum membrane^48^. The fact that SPTLC2 and SPTLC3, when expressed individually in Hek293 cells, increase SPT activity^4^ indicates that both subunits can act independently. However, SPTLC3 mRNA was undebatable in both *Sptlc2* KO and control colons, although SPTLC1 and SPTLC2 could be easily detected (Supplementary Figure S7). The lethality of intestine-specific *Sptlc2* deficiency clearly indicates that SPTLC2 is responsible for SPT activity in the intestine.

In conclusion, sphingolipid *de novo* synthesis is crucial for intestinal cell survival and barrier function. The blockage of SPT activity in the intestine causes intestinal cell death and impairment of barrier function.

## Materials and methods

### Generation of inducible intestine-specific *Sptlc2* KO mice

*Sptlc2-*Flox mice were crossed with Villin-Cre-ER^T2^ (estrogen receptor T2), which yielded *Sptlc2*-Flox/Villin-Cre-ER^T2^ mice (Supplemental Fig. 1A). Mice were genotyped using PCR (Supplemental Fig. 1B). To delete *Sptlc2* in the intestine, tamoxifen (Sigma Aldrich; 0.5 mg per mouse, dissolved in 200 μl of corn oil) was injected intraperitoneally every day for three times total. Controls were *Sptlc2*-Flox mice that had also been injected with tamoxifen. Both male and female mice (10 weeks old) with a C57BL/6J background were used. All experiments were approved by the Institutional Animal Care and Use Committee of the State University of New York Downstate Medical Center and conformed with the “Guide for the Care and Use of Laboratory Animals” published by the US National Institute of Health (NIH publication No. 85-23, revised 1996).

To rescue the lethality of *Sptlc2* KO male and female mice, we treated the mice with low- absorption antibiotics (25 mg/kg vancomycin; 20 mg/kg gentamycin; 5 mg/kg rifampicin) via oral gavage and with dexamethasone (0.8 mg per mouse, intramuscular) 2 weeks before tamoxifen treatment (see above) and continued for total 30 days. Thereafter, the mice were used for the assays described below.

### Isolation of plasma membrane from primary enterocytes

Primary enterocytes were isolated according to previous reports^49^. Plasma membrane was isolated from primary enterocytes according to our published protocol^50^.

### Sphingolipid analyses

Sphingomyelin, ceramide, and sphingosine-1-phosphate levels were measured in control and *Sptlc2* KO enterocyte plasma membrane by liquid chromatography–coupled tandem mass spectrometry, as described^34^.

### FPLC

Plasma lipid distribution was quantified by FPLC using a Superose 6B column. A 250-μl aliquot of pooled plasma was injected onto the column and eluted with 50 mM Tris-HCl (pH 7.4) at a constant flow rate of 0.35 ml/min. A 100-µl aliquot from each fraction was used for the measurement of lipids.

### Gene expression analysis by real-time PCR

Mice were sacrificed by cervical dislocation. The jejunum, liver, and kidneys were dissected and total RNA extracted from each tissue using the RNeasy Mini kit (Qiagen). cDNA was synthesized with an Invitrogen Superscript^TM^ ІІІ First-strand Synthesis kit. PCR was performed in a volume of 20 μl with the SYBR Green kit from Applied Biosystems. The 18S rRNA was used as the internal control. The amplification program was: 95 °C for 10 min, followed by 40 amplification cycles of 95 °C for 15 s and 60 °C for 1 min. Each sample was in triplicate. Relative gene expression is presented as mean ± SD. Mouse *Sptlc2* primers were: forward, AGCCATTTCCGGTTTCGGAGA; reverse, TCCTCAAGTACCCGTTCCTCA. Mouse 18S rRNA primers were: forward, AGTCCCTGCCCTTTGTACACA; reverse, GATCCGAGGGCCTCACTAAAC.

### Western blotting

Primary enterocyte homogenates were subjected to western blotting as described^51^ with antibodies against NPC1L1 (a gift from Dr. Bao Liang Song, Chinese Academy of Sciences), CD36 (Abcam), ATP-binding cassette transporter 8 (ABCG8; Novus Biologicals), and ABCA1 (Novus Biologicals). Homogenates of small intestine and colon tissue in PBS were used to determine levels of SPTLC2 (Proteintech Group) and SPTLC1 (BD Transduction). Colon tissue homogenate was used for western blotting for mucin2 (antibody from Novus Biologicals) and cleaved caspase-3 (antibody from Cell Signaling Technology). The activation of the MAPK pathway in colon was assessed using phospho-specific antibodies for ERK, p38, and JNK (Cell Signaling Technology, Cat. #9910S). Total protein of ERK, p38, and JNK was also quantified in colon (Cell Signaling Technology, Cat. #9926S). GAPDH was used as a loading control.

### SPT activity measurement

Large intestine and small intestine from intestine-specific *Sptlc2* KO and wild type mice were homogenized in SPT homogenization buffer (10 mM HEPES, pH 7.5, 10 mM Sucrose, 1 mM EDTA), and centrifugation at 4500 rpm at 4 °C for 15 min. The supernatant was adjusted to a final concentration of 0.25 M sucrose, and then spin at 50,000 rpm (SW55) for 1 h at 4 °C. The microsome pellet was resuspended in SPT microsome suspension buffer (10 mM HEPES, pH 7.5, 0.25 M Sucrose) for SPT activity assay.

SPT activity was assayed in 200 µl of the reaction system containing 50 mM HEPES, pH 7.5, 5 mM EDTA, 5 mM DTT, 50 µM Pyridoxal phosphate, 200 µM Palmitoyl-CoA, 400 µM Serine, and 0.5 µCi ^14^C-serine. The reaction was initiated by the addition of microsomal protein, incubate at 37 °C for 10 min in a water bath, and stopped by adding 400 µl of 0.5 N NH_4_OH. The lipids were extracted by adding 800 µl of chloroform/methanol (2:1, v/v) and mixing vigorously. The organic phase was collected after centrifugation (6000×*g*) for 10 min and dried under nitrogen gas. Lipids were dissolved in 20 µl of chloroform and then were applied to TLC plate (chloroform/methanol/20% ammonia, 14/6/1, v/v). The TLC plate was exposed to a phosphor screen and then scanned on a phosphor imager.

### H&E staining

The small intestine and colon were dissected and fixed overnight in 4% formalin. The tissue was embedded in paraffin and then sliced (5 μm thick). Each slice was deparaffinized and stained with H&E.

### Immunofluorescence staining and immunohistochemistry

Intestinal tissues were fixed in 4% formalin overnight at 4 °C before preparation of 5-µm paraffin sections. Prior to antibody staining, the sections were deparaffinized in xylene and rehydrated in a gradient series of ethanol, then subjected to high-temperature antigen retrieval in 50 mM Tris-HCl (pH 9.0), 1 mM EDTA. Sections were permeabilized and blocked in Tris-HCl with 0.5% Triton X-100 and 5% horse serum. The sections were incubated overnight at 4 °C with the primary antibodies rabbit anti-NPC1L1, goat anti-villin (Santa Cruz Biotechnology), and rabbit anti-Ki67 (Millipore). Slides were then incubated with fluorescently labeled secondary antibodies. Intestinal tissues fixed in 4% formalin were sent to Histowiz Inc. (SUNY Biotechnology Incubator) for immunohistochemical analysis of cleaved caspase-3 and for TUNEL staining.

### Periodic acid-schiff staining and Alcian blue staining

Deparaffinized and rehydrated intestinal tissue sections were treated with periodic acid (Abcam) for 5 min at room temperature. Slides were washed in distilled water and then stained with Schiff’s reagent (Abcam) for 15 min at room temperature, followed by a 5-min wash in running tap water. The sections were then counterstained with hematoxylin, washed in running tap water for 2 min, dehydrated twice in 95% ethanol and twice in 100% ethanol, and cover slipped. Proximal colon samples fixed in 4% formalin were sent to Histowiz Inc. for Alcian Blue staining.

### Measurement of LPS

Plasma samples (50 µl) were sent to Assaygate Inc. (Maryland) for bacterial LPS measurement.

### Information on human samples

We obtained paraffin embedded human colon disease tissue array from US Biomax, Inc. (Cat# BC05002a and CO809a). Colons from Crohn’s disease, chronic colitis, ulcerative colitis, and controls were stained by SPTLC2 antibody and by H&E. We obtained human Crohn’s and colitis tissue qPCR array from QriGene Technologies, Inc. (Cat# CCRT101), and then we did real-time PCR for SPTLC2 mRNA.

### Statistical analysis

Data are expressed as mean ± SD. Data between two groups were analyzed with the unpaired, two-tailed Student’s *t*-test, and among multiple groups with analysis of variance followed by the Student-Newman-Keuls test. Statistical significance was set as *P* < 0.05.

## Electronic supplementary material

Supplement
