# Supplementary material for: Sphingolipid de novo biosynthesis is essential for intestine cell survival and barrier function
Source: Cell Death Dis. 2018 Feb 7;9(2):173. doi: 10.1038/s41419-017-0214-1 (PMC5833386; doi:10.1038/s41419-017-0214-1)

Supplement

Supplementary Figure S1
Generation of intestine-specific inducible *Sptlc2* KO mice. (A) Strategy used to disrupt mouse Sptlc2 in the intestine. (B) Mouse genotyping.

Supplementary Figure S2
Effect of *Sptlc2* deficiency on body weight, food consumption, and body temperature. (A) Body weight. (B) Average food consumption. (C) Body temperature at day 6 after tamoxifen treatment. Values represent the mean ± SD, n = 8, *P < 0.05. I-Sptlc2 KO, intestine-specific Sptlc2 knockout.

Supplementary Figure S3
Effect of *Sptlc2* deficiency on mRNA levels on ORMDL1-3 as well as ssSPTa and ssSPTb. Values represent the mean ± SD, n = 5, *P < 0.01. I-Sptlc2 KO, intestine-specific Sptlc2 knockout.

Supplementary Figure S4
Immunostaining of lymphocytes in *Sptlc2* deficient and control colons. (A) CD4 T cell staining. (B) B220 B cell staining. Red arrows indicate positive staining. I-Sptlc2 KO, intestine-specific Sptlc2 knockout.

Supplementary Figure S5
Effect of *Sptlc2* deficiency in cholesterol absorption. Sptlc2-Flox/Villin–Cre-ERT2 and control male mice were treated with tamoxifen. At day 6 post-treatment, the mice were gavaged with 0.1 μCi [14C] cholesterol and 0.2 μCi [3H] sitostanol in 20 μL olive oil. Feces were collected at 48 h post-treatment, and lipids were extracted for counting. Values represent the mean ± SD, n = 7, *P < 0.01.

Supplementary Figure S6
Effect of *Sptlc2* deficiency on spleen, blood cells, and lung. (A) Mousspleen weight and splenocyte number. (B) Blood cell counts. (C) Low- (top) and high- (bottom) magnification images of mouse lung. Images are representative of five fields. Values represent the mean ± SD, n = 5, *P < 0.01. CON, control; KO, intestine-specific Sptlc2 knockout.

Supplementary Figure S7
SPTLC1, 2 and 3 mRNA measurement in I-Sptlc2 KO and control mouse small and large intestines. Real-time PCR was performed. (A) small intestine. (B) large intestine. Values represent the mean ± SD, n = 3, *P < 0.001

Supplementary Fig. S1. Li et al.

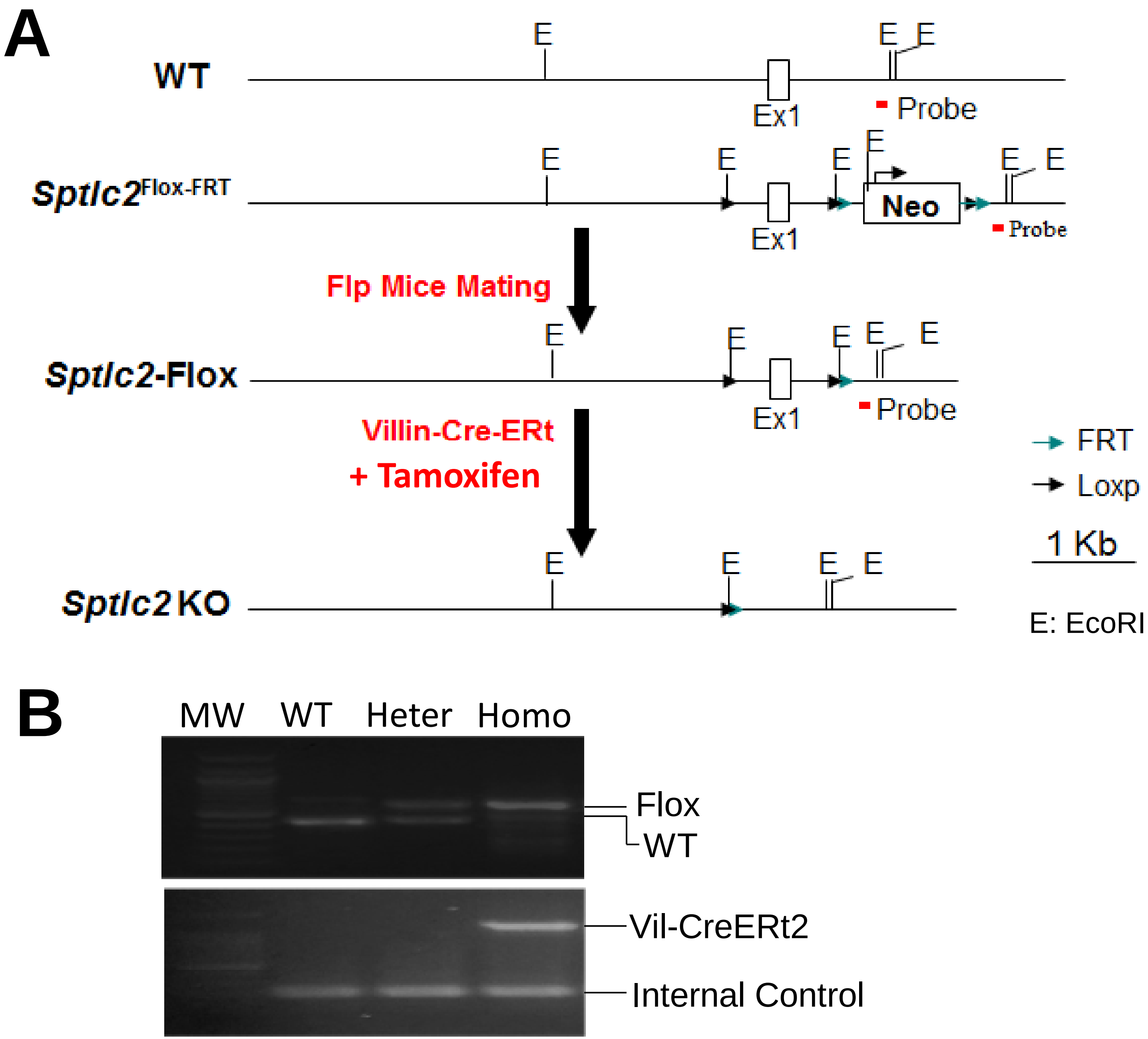

Supplementary Fig. S2. Li et al.

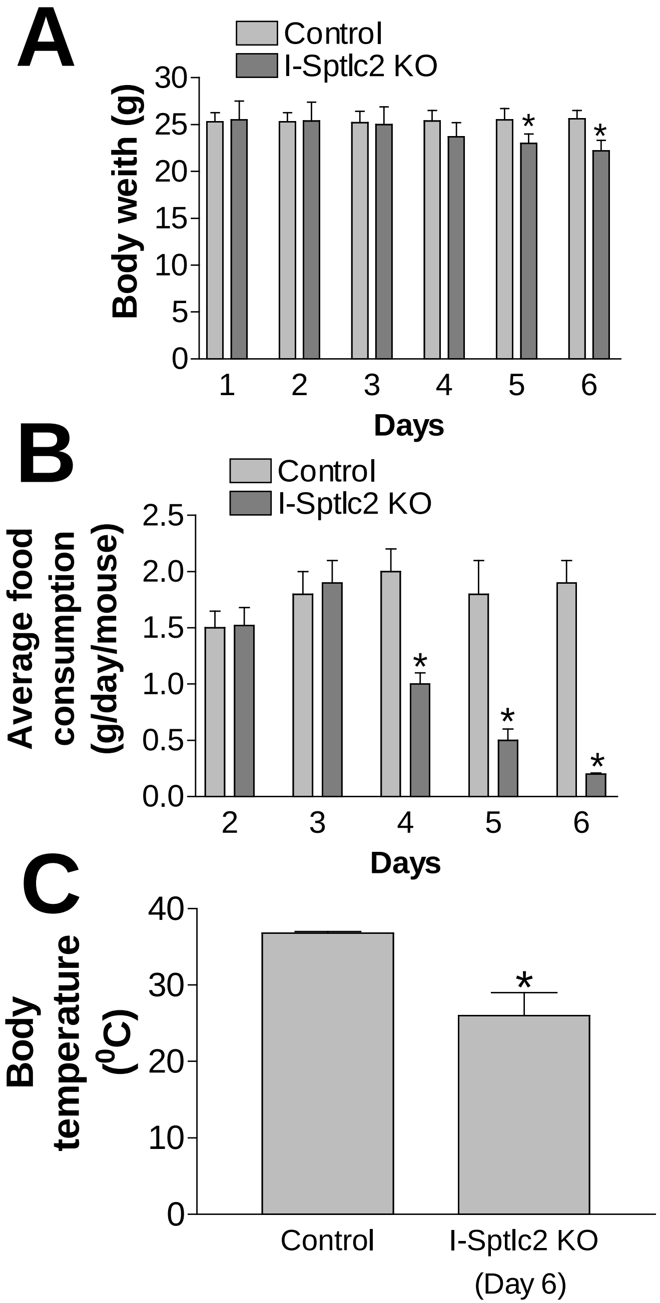

Supplementary Fig. S3. Li et al.

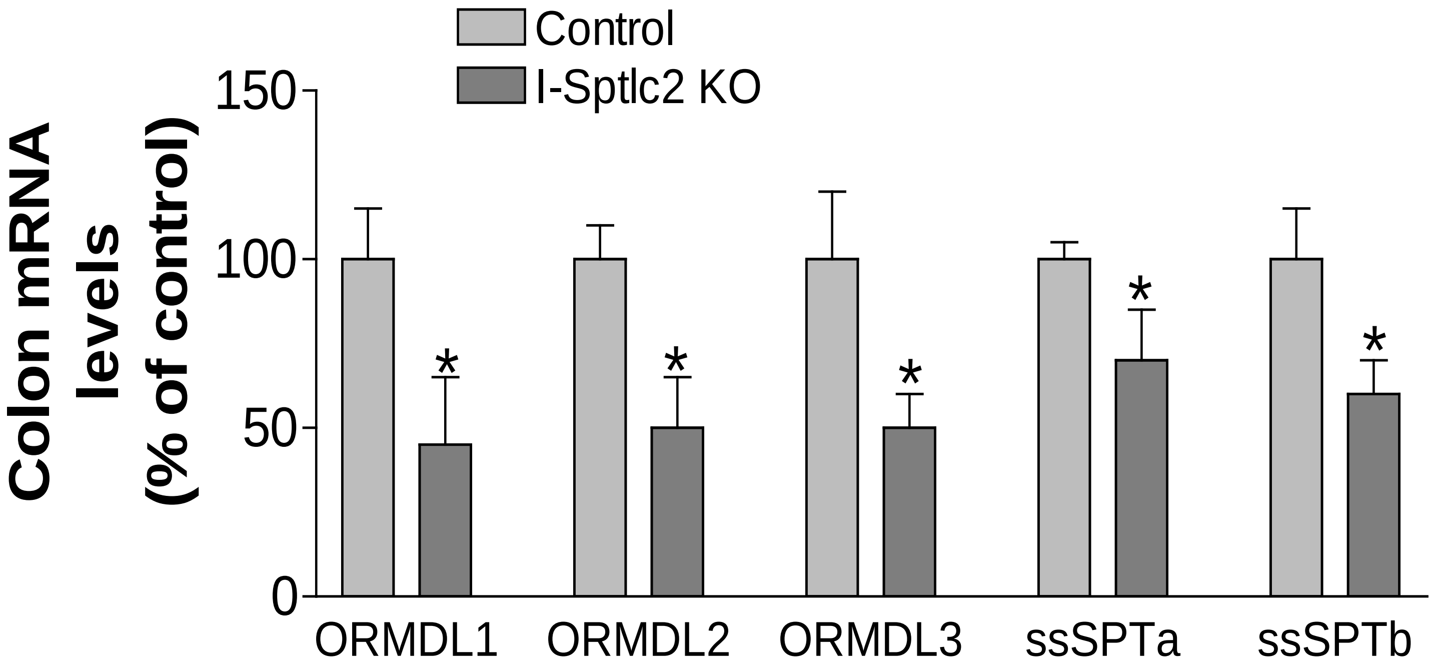

Supplementary Fig. S4. Li et al.

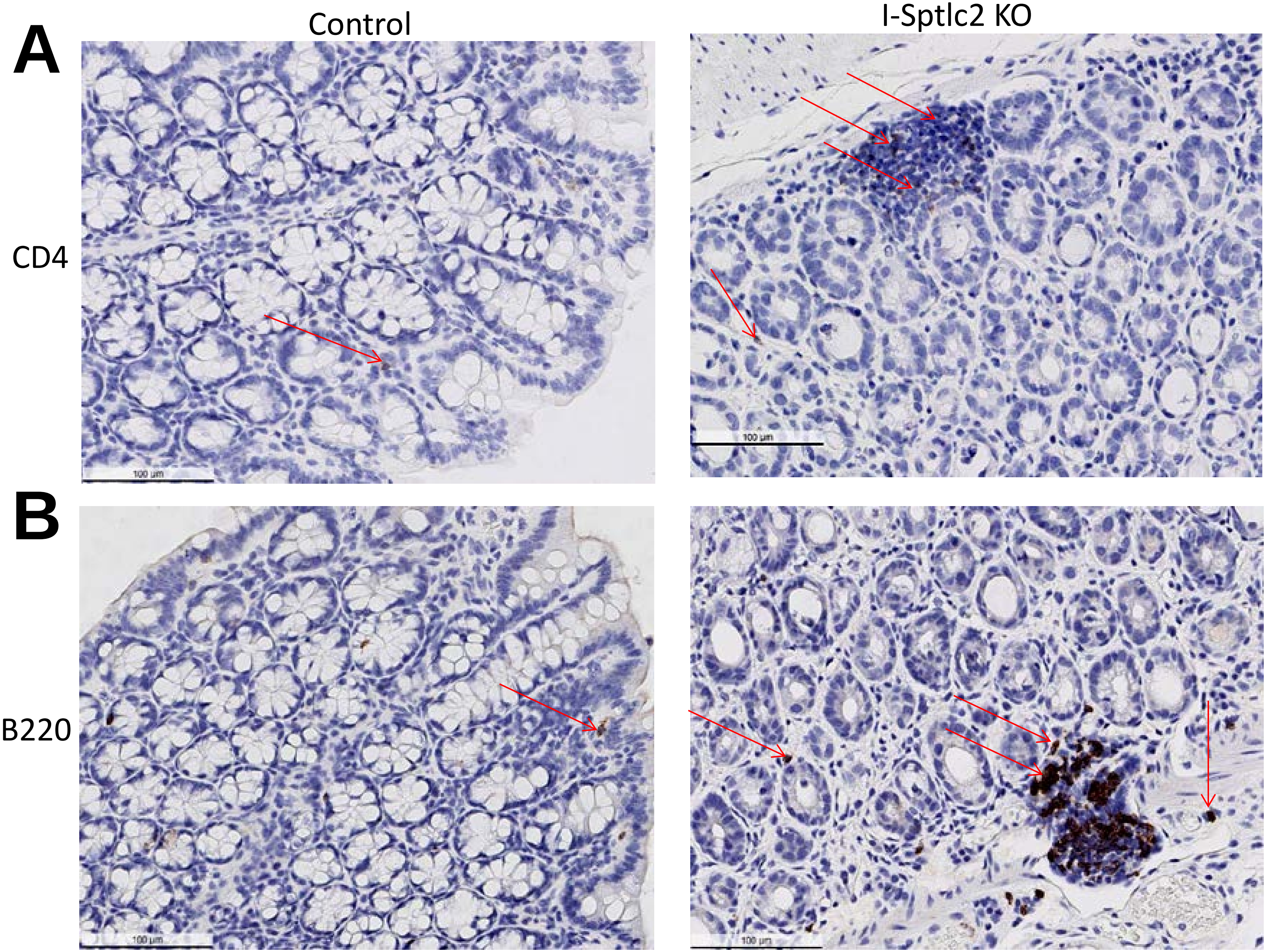

Supplementary Fig. S5. Li et al.

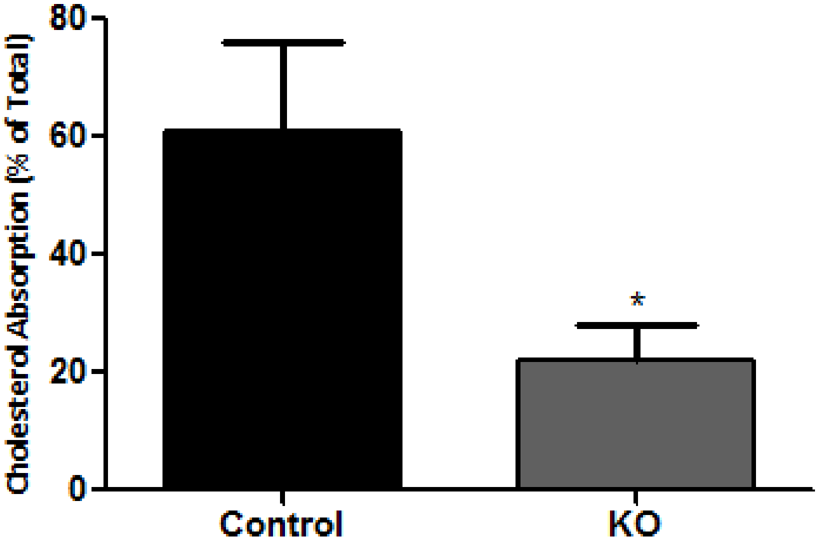

Supplementary Fig. S6. Li et al.

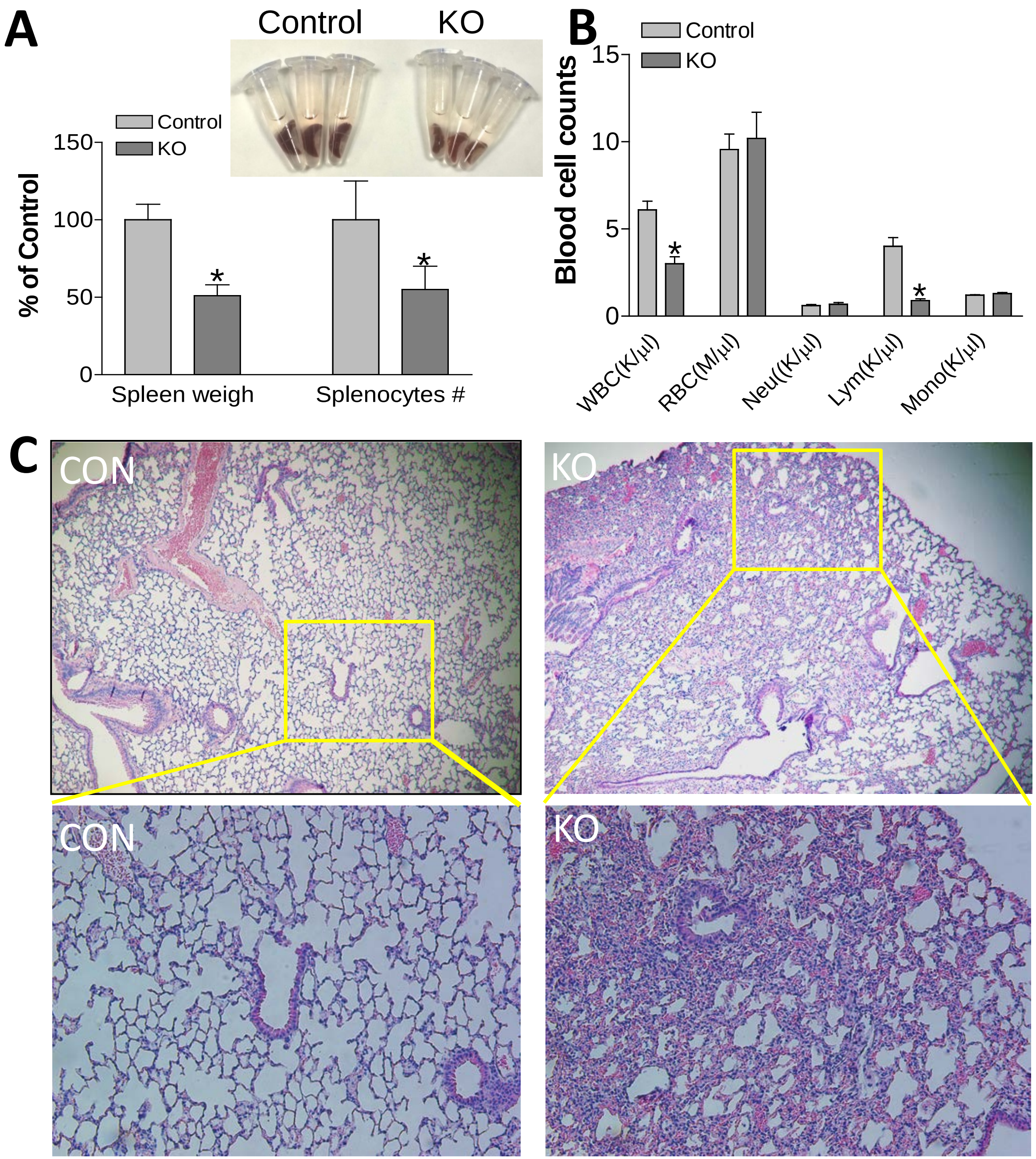

Supplementary Fig. S7

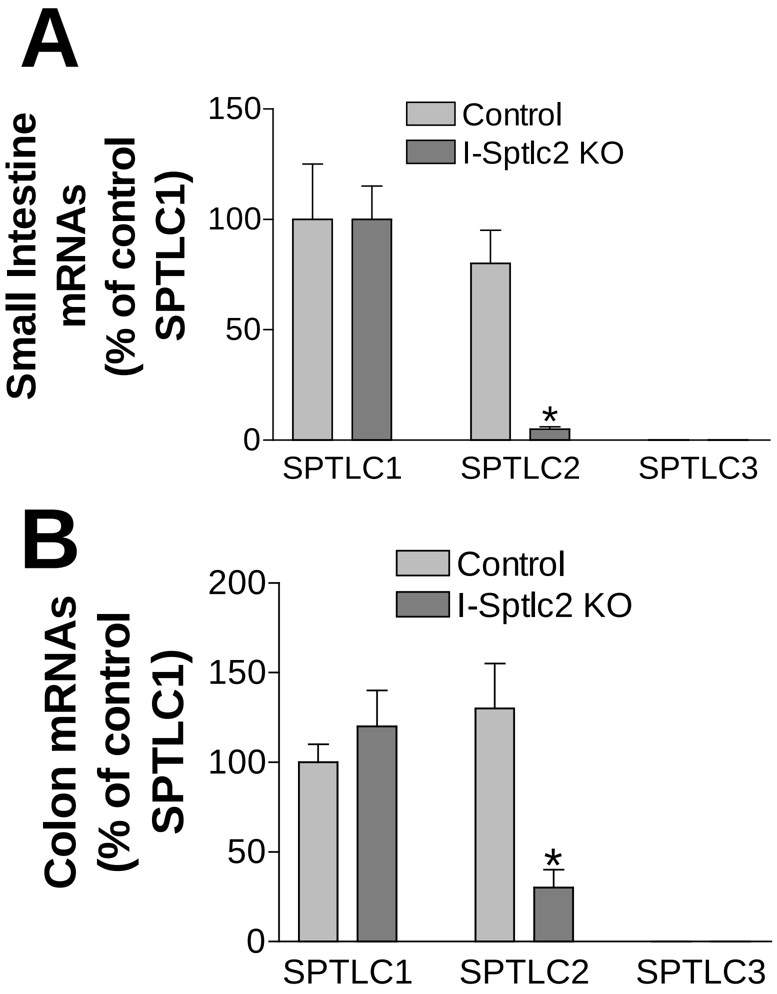

Supplement: Supplementary file 1 — Supplement [file 41419_2017_214_MOESM1_ESM.pdf]
